# The Associations among Insulin Resistance, Hyperglycemia, Physical Performance, Diabetes Mellitus, and Cognitive Function in Relatively Healthy Older Adults with Subtle Cognitive Dysfunction

**DOI:** 10.3389/fnagi.2017.00072

**Published:** 2017-03-23

**Authors:** Hiroyuki Umegaki, Taeko Makino, Kazuki Uemura, Hiroyuki Shimada, Takahiro Hayashi, Xian Wu Cheng, Masafumi Kuzuya

**Affiliations:** ^1^Department of Community Healthcare and Geriatrics, Nagoya University Graduate School of MedicineNagoya, Japan; ^2^Institute of Innovation for Future Society, Nagoya UniversityNagoya, Japan; ^3^Liberal Arts and Sciences, Faculty of Engineering, Toyama Prefectural UniversityToyama, Japan; ^4^Department of Preventive Gerontology, Center for Gerontology and Social Science, National Center for Geriatrics and GerontologyObu, Japan

**Keywords:** glycihemoglobine, HOMA-IR, gait speed, sarcopenia, executive function, memory

## Abstract

Insulin resistance (IR), diabetes mellitus (DM), sarcopenia, and cognitive dysfunction are thought to be mutually associated. We conducted a comprehensive assessment of the relationships among IR, gait speed, hyperglycemia, and DM by cross-sectionally analyzing the baseline data of an interventional study for cognitive preservation with physical exercise (the TOyota Preventional Intervention for Cognitive decline and Sarcopenia [TOPICS]). The participants (*n* = 444) were relatively healthy older individuals who had mild cognitive impairment without dementia, and 61 of the participants had DM. Slow gait speed and hyperglycemia were associated with cognitive dysfunction, mainly in the executive function domain, whereas IR was associated with memory impairment. The participants with DM had lower general cognition and executive function. Executive dysfunction in the DM participants seemed to be partly explained by hyperglycemia and/or slow gait speed. Our findings confirmed that IR, DM, sarcopenia, and cognitive dysfunction are mutually associated in complex ways. Understanding the mechanisms underlying these associations will lead to effective strategies to prevent and treat cognitive dysfunction in older individuals.

## Introduction

Along with the increasingly aging populations in mainly developed countries, the number of individuals with cognitive impairment has grown, leading to increases in medical expenses as well as economic, physical, and psychological care-related burdens. There are no curative treatments for cognitive dysfunction at this time, and the prevention of cognitive decline is a critical issue. Toward this end, the identification of cognitive dysfunction risk factors (especially modifiable factors) is urgently needed, and interventional trials for such risk factors would be warranted in order to develop effective therapies.

Many studies have shown that individuals with diabetes mellitus (DM) have higher risks of dementia and cognitive dysfunction (Koekkoek et al., 2015). The mechanism that underlies DM-related cognitive dysfunction has not been fully elucidated, but several hypotheses have been put forward (Umegaki, 2014). Hyperglycemia is involved in the mechanism of DM-associated cognitive dysfunction (Geijselaers et al., 2015), as are other factors. Insulin resistance (IR) often leads to DM, and patients with DM sometimes have cognitive dysfunction. The results obtained in basic researches have suggested that systemic IR may induce brain IR, or a deficiency of insulin effects in the brain, which in turn may lead to the phosphorylation of tau and the production of amyloid beta, and to Alzheimer disease (AD)-related pathology (Bedse et al., 2015).

Muscle is a major target of insulin. Insulin binds to the receptors on the muscle cells to transport blood glucose into muscle (Ryder et al., 2001). In humans, muscle mass and performance decrease with age, via a phenomenon known as sarcopenia (Rosenberg, 1997). Sarcopenia is thus characterized by a loss of muscle mass and strength (Cruz-Jentoft et al., 2010). Because insulin has a catabolic effect, IR, which induces impairment of protein synthesis in muscle, is suspected to be involved in the development of sarcopenia (Umegaki, 2016). The diagnosis of sarcopenia is based on the reduction of muscle mass, but a loss in muscle power and a decline in physical performance are also required for the diagnosis.

Gait speed is often used as an index of physical performance. Several studies have reported that slower gait speed was associated with cognitive dysfunction, mainly in the executive function domain (Kearney et al., 2013; Demnitz et al., 2016), and that slower gait speed is predictive of adverse health events including dementia (Buchner et al., 1996; Guralnik et al., 2000). The age-associated muscle mass reduction in sarcopenia has also been reported to be associated with cognitive dysfunction (Nourhashémi et al., 2002; Doi et al., 2014). Moreover, Burns et al. (2010) reported that the age-associated reduction of lean muscle mass is accelerated in AD and is associated with brain atrophy and cognitive performance. Several studies have reported that DM is associated with sarcopenia or low muscle performance (Kearney et al., 2013; Demnitz et al., 2016). It may be speculated that DM-related cognitive dysfunction is at least partly associated with sarcopenia or low muscle performance, although there have been few studies examining these potential associations.

DM is often associated with various metabolic alterations, such as hyperglycemia and IR, as well as with indices of sarcopenia, particularly low muscle performance (Umegaki, 2016; Zaslavsky et al., 2016). Both sarcopenia (Nourhashémi et al., 2002; Doi et al., 2014) and IR (Umegaki et al., 2008; Ekblad et al., 2015) have been associated with cognitive dysfunction in older subjects. Therefore, the underlying mechanisms of DM-related cognitive dysfunction may include or be associated with IR and low muscle function. However, the contributions of sarcopenia and IR to DM-related cognitive dysfunction have not been studied extensively. Thus, while it is anticipated that associations exist among IR, sarcopenia, and cognitive dysfunction, few studies have comprehensively assessed the associations these associations. In the present study we investigated the associations among IR, sarcopenia, and cognitive dysfunction to determine the contributions of IR and declining physical performance to the development of DM-related cognitive dysfunction.

## Methods

This was a randomized control trial to assess the effects of different types of exercise (aerobic, resistance, or both) on cognition in older subjects with a slight decline of memory function based on the cross-sectional analysis of baseline data from the TOyota Preventional Intervention for Cognitive decline and Sarcopenia [TOPICS].

The study protocol was approved by our University's Ethics Committee (Graduate School of Medicine, Nagoya University; approval no. 2014-0155-2) and registered with the University Hospital Medical Information Network (UMIN) clinical trials registry (no. UMIN000014437). Written informed consent was obtained from all participants prior to their inclusion in the study.

### Participants

Participants were screened with the use of a questionnaire comprised of 25 self-completed items (Arai and Satake, 2015), including three items concerning subjective cognitive decline: Q18, Do your family or friends point out your memory loss? (e.g., “You ask the same question over and over again”); Q19, Do you make a call by looking up phone numbers?; and Q20, Do you find yourself not knowing today's date? Respondents who answered yes to Q18 or Q20 or who answered no to Q19 were regarded as being at high risk of cognitive decline (Maki et al., 2012). The questionnaire was mailed to community-dwelling residents aged 65–85 years in selected areas in the city of Toyota, Aichi, Japan. Residents who met the criteria for being at risk of cognitive decline according to at least one of the three questionnaire items described above were recruited via a mailing describing the interventional study project.

The exclusion criteria were as follows: the subject (1) meets clinical criteria for dementia, (2) has any disability with respect to basic and instrumental activities of daily living (ADLs), (3) requires support or care from the Japanese public long-term care insurance system, (4) has a Mini-Mental State Examination (MMSE) score ≤19, (5) has severe visual impairment, (6) has been diagnosed with a neurodegenerative disease (e.g., Parkinson's disease), (7) has medical conditions for which exercise is contraindicated, (8) has a psychiatric disease (i.e., major depressive disorder), or (9) has a history of serious cardiovascular, musculoskeletal, respiratory, or cerebrovascular disease or other severe health issue.

DM was defined as a self-report of diagnosed DM, current use of antidiabetic medication, fasting plasma glucose ≥126 mg/dl, or HbA1c ≥6.5%. We excluded fasting blood glucose >140 mg/dl from the present analysis because it is not suitable for the assessment of IR in these subjects (Wallace et al., 2004) with homeostatic model assessment-insulin resistance (HOMA-IR; Matthews et al., 1985).

### Measurements

A battery of neuropsychological assessments were performed by a group of well-trained clinical psychologists, speech therapists, and occupational therapists. Each score was rechecked by a single neuropsychologist who was blinded regarding the other data associated with the participants. The following tests were used. To assess memory, we used the Logical Memory II subtest of the WMS-R (Guo et al., 2009; Aisen et al., 2010); the MSE for general cognitive ability (Folstein et al., 1975); and the Logical Memory I subtests and Visual Reproduction I and II subtests of the WMS-R (Wechsler, 1987). For verbal fluency, we used the category fluency (animal naming) test and the letter fluency test. For working memory, we used the Digit Span (forward and backward) subtest and Visual Memory Span (forward and backward) subtest of the WMS-R for working memory. Finally, to assess processing speed, we used the Digit Symbol subtest of the Wechsler Adult Intelligence Scale-III (WAIS-III; Wechsler, 1997); and the Trail Making Test parts A and B (Spreen and Strauss, 1998).

### Physical function

We measured grip strength in the dominant hand of each participant with the use of a portable grip strength dynamometer (GRIP-D; Takei, Ltd., Niigata, Japan). Gait speed was assessed during normal walking, with the participant instructed to walk at his or her preferred speed (Doi et al., 2014).

### Body composition

We used a bioelectrical impedance data acquisition system (Inbody 430; Biospace Co., Seoul, Korea) to perform the bioelectrical impedance analyses. This system uses electrical current at three frequencies (5, 50, 250 kHz) to directly measure the amounts of extracellular and intracellular water in the human body. Using segmental body composition and muscle mass, each participant's appendicular skeletal muscle mass was determined and retained for further analysis. The skeletal muscle mass index (SMI) was calculated by dividing the participant's muscle mass by his or her height in meters squared (kg/m^2^).

### Blood markers

A blood sample was obtained after the participant had fasted for ≥12 h. Serum samples were analyzed for total cholesterol, high-density lipoprotein cholesterol, triglyceride, glucose, glycosylated hemoglobin (HbA_1c_), and insulin. The apolipoprotein E (apoE) genotypes were classified into e2/e2, e2/e3, e3/e3, e2/e4, e3/e4, and e4/e4 based on the immunoblot technique using isoelectric focusing. We obtained the blood samples in the morning (between 9:30 a.m. and 12:00 p.m.). Insulin resistance was assessed by HOMA-IR (Matthews et al., 1985).

### Statistical analysis

Student's *t-*test was used for the comparison of continuous variables. The distribution of frequencies for categorical variables was analyzed with the Chi-square (χ^2^) test. We performed a multiple regression analysis to investigate the associations of cognitive performance and the factors of HOMA-IR values, gait speed, HbA1c, and DM diagnosis with adjustment for age, sex, number of school years, and apoE4 status. In the tests that revealed a significant association with DM, we performed further adjustment for any factors that were significantly associated with cognition among HOMA-IR values, gait speed, and HbA1c.

## Results

Table 1 shows the background characteristics of the participants. In both males and females, the body mass index (BMI) values were significantly larger in the participants with DM. Among the participants with DM (*n* = 61), the blood glucose, HbA1c, insulin, and HOMAR-IR values were higher, but total cholesterol (T-chol) and high-density lipoprotein cholesterol (HDL-chol) were lower compared to those in the non-DM group (*n* = 383).

**Table 1:** **Background characteristics of the participants**.

	**Total**	**DM (−)**	**DM (+)**	***p*-value**
No. of participants	444	383	61	
Age (yrs)	72.4 ± 4.7	72.4 ± 4.7	72.7 ± 4.8	0.651
Sex	52.3(232)	50.7(194)	62.3(38)	0.06
Education (yrs)	11.4 ± 2.4	11.5 ± 2.4	11.4 ± 2.8	0.905
BMI total (Kg/m^2^)	22.8 ± 2.9	22.6 ± 2.8	24.2 ± 3.2	<0.001
BMI male (Kg/m^2^)	23.2 ± 2.8	22.9 ± 2.5	24.4 ± 3.3	0.002
BMI female (Kg/m^2^)	22.5 ± 3.0	22.3 ± 3.0	23.8 ± 3.1	0.035
sBP (mmHg)	155.0 ± 20.2	154.9 ± 20.5	155.9 ± 18.4	0.711
dBP (mmHg)	79.2 ± 12.1	79.5 ± 12.1	77.3 ± 11.5	0.177
Tchol (mg/dl)	212.8 ± 36.9	216.6 ± 35.9	189.2 ± 34.7	<0.001
HDL chol (mg/dl)	66.5 ± 17.6	67.5 ± 17.3	60.3 ± 18.3	0.003
TG (mg/dl)	108.2 ± 57.0	107.5 ± 56.4	112.9 ± 61.3	0.489
FBS (mg/dl)	99.4 ± 12.5	97.1 ± 10.3	114.3 ± 14.6	<0.001
HbA1c (%)	5.8 ± 0.5	5.7 ± 0.3	6.6 ± 0.5	<0.001
Insulin (μU/ml)	5.6 ± 8.4	5.2 ± 5.8	8.0 ± 17.4	0.017
BUN (mg/dl)	15.6 ± 4.3	15.5 ± 4.1	16.3 ± 5.3	0.151
sCr (mg/dl)	0.77 ± 0.23	0.76 ± 0.22	0.84 ± 0.30	0.009
Hb (g/dl)	13.9 ± 1.3	14.0 ± 1.3	13.7 ± 1.1	0.204
CRP(mg/dl)	122 ± 349	120 ± 354	131 ± 320	0.819
HOMA-IR	1.43 ± 2.55	1.29 ± 1.52	2.38 ± 5.74	0.002
ApoE4 carrier [%(N)]	20.5 (91)	21.4 (82)	14.8 (9)	0.305

*The data are the mean ± SD. Student's t-test and the χ^2^ test were used to study the differences between the participants with DM and the non-DM participants. BMI, body mass index; sBP, systolic blood pressure; dBP, diastolic blood pressure; Tcho, total cholesterol; HDL chol, high-density lipid cholesterol; TG, triglyceride; FBS, fasting blood glucose; HbA1c, glycated hemoglobin A1c; BUN, blood urea nitrogen; sCr, serum creatinine; Hb, hemoglobin; CRP, C-reactive protein; HOMA-IR, homeostatic model assessment-insulin resistance; ApoE, apolipoprotein E. In the DM group the values of blood glucose, HbA1c, insulin, and HOMAR-IR were higher, but T-chol and HDL-chol were lower compared to those in the non-DM group*.

The body compositions and performance of the DM and non-DM groups were comparable, with the exception that gait speed was significantly lower (*p* = 0.024) in the DM group (Table 2).

**Table 2:** **Body compositions of the participants**.

	**Total**	**DM (−)**	**DM (+)**	***p*-value**
	***n* = 444**	***n* = 383**	***n* = 61**	
**SMI**				
Male	7.2 ± 0.6	7.1 ± 0.6	7.3 ± 0.6	0.118
Female	5.7 ± 0.6	5.7 ± 0.6	5.9 ± 0.7	0.160
**APPENDICULAR MUSCLE MASS**				
Male	19.2 ± 2.4	19.1 ± 2.4	19.6 ± 2.2	0.201
Female	13.0 ± 1.8	12.9 ± 1.8	13.3 ± 20.	0.423
**GRIP STRENGTH**				
Male	33.9 ± 5.6	34.0 ± 5.7	33.2 ± 5.1	0.441
Female	21.7 ± 4.1	21.9 ± 4.0	20.1 ± 5.1	0.053
**GAIT SPEED**				
	1.41 ± 0.21	1.42 ± 0.20	1.36 ± 0.21	0.024

*The data are the mean ± SD. Student's t-test and the χ^2^ test were used to study the differences between the participants with DM and the non-DM participants. SMI, skeletal muscle mass index. Gait speed was significantly lower in the DM group*.

In the cognitive assessment, the scores of MMSE, semantic fluency, digit span, digit symbol, TMT-A, and TMT-B were significantly lower in the DM group (Table 3).

**Table 3:** **Cognitive function of the participants**.

	**Total**	**DM (−)**	**DM (+)**	***p-*value**
	***n* = 444**	***n* = 383**	***n* = 61**	
MMSE	26.2 ± 2.7	26.6 ± 2.7	25.4 ± 2.4	0.022
Logical memory I	14.7 ± 6.3	14.7 ± 6.4	14.7 ± 6.2	0.991
Visual reproduction I	32.1 ± 6.1	32.2 ± 6.1	31.4 ± 6.0	0.342
Logical memory II	10.0 ± 6.2	10.1 ± 6.4	9.6 ± 5.3	0.569
Visual reproduction II	22.4 ± 10.0	22.5 ± 10.1	22.2 ± 9.4	0.874
Letter fluency	8.7 ± 3.4	8.7 ± 3.4	8.7 ± 3.5	0.972
Semantic fluency	16.2 ± 4.3	16.5 ± 4.4	14.8 ± 3.4	0.004
Digit span	11.1 ± 2.8	11.3 ± 2.8	10.4 ± 2.7	0.037
Digit symbol	11.1 ± 2.7	11.3 ± 2.6	10.1 ± 3.0	0.001
TMT-A	45.4 ± 16.3	44.7 ± 15.4	49.9 ± 20.1	0.020
TMT-B	127.3 ± 58.0	124.1 ± 55.3	147.9. ± 70.2	0.004
GDS-15	4.07 ± 2.86	4.02 ± 2.91	4.39 ± 2.72	0.347

*The data are the mean ± SD. Student's t-test and the χ^2^ test were used to study the differences between the participants with DM and the non-DM participants. MMSE, Mini-Mental State Examination; TMT, Trail Making Test; GDS-15, Geriatric Depression Scale-15. The values of MMSE, semantic fluency, digit span, digit symbol, TMT-A, and TMT-B were significantly lower in the DM group*.

The results of the multiple regression analysis (Table 4) showed that HOMA-IR was associated with logical memory II after adjustment for age, sex, school years, and apoE4 status. Gait speed was associated with logical memory I, semantic fluency, letter fluency, digit symbol, TMT-A, and TMT-B. The participants' HbA1c values were associated with semantic fluency, digit symbol, TMT-A, and TMT-B. We also observed that DM was associated with MMSE, semantic fluency, digit span, digit symbol, TMT-A, and TMT-B.

**Table 4:** **Results of a multiple regression analysis of the associations between neurocognitive assessments and HOMA-IR, gait speed, HbA1c, and DM**.

	**HOMA-IR**	**Gait speed**	**HbA1c**	**DM**
MMSE	−0.026 (0.577)			
		0.043 (0.377)		
			−0.034 (0.463)	
				−0.105 (0.022)
Logical memory 1	−0.024 (0.602)			
		0.104 (0.034)		
			−0.001 (0.983)	
				0.009 (0.847)
Logical memory 2	−0.091 (0.047)			
		0.088 (0.069)		
			−0.032 (0.492)	
				−0.017 (0.720)
Visual reproduction1	−0.015 (0.745)			
		0.028 (0.555)		
			−0.011 (0.809)	
				−0.047 (0.296)
Visual reproduction2	−0.063 (0.161)			
		−0.031 (0.509)		
			−0.012 (0.784)	
				−0.005 (0.915)
Semantic fluency	−0.054 (0.260)			
		0.132 (0.008)		
			−0.168 (0.000)	
				−0.146 (0.002)
		0.119 (0.017)	−0.129 (0.031)	0.054 (0.370)
Letter fluency	−0.067 (0.150)			
		0.108 (0.027)		
			−0.046 (0.325)	
				0.013 (0.771)
Digit symbol	−0.063 (0.171)			
		0.225 (0.000)		
			−0.102 (0.027)	
				−0.149 (0.001)
		0.211 (0.000)	−0.022(0.702)	−0.114 (0.047)
Digit span	−0.063 (0.178)			
		0.095 (0.053)		
			−0.033 (0.473)	
				−0.095 (0.041)
Visual memory span	−0.046 (0.318)			
		0.071 (0.143)		
			−0.027 (0.553)	
				−0.060 (0.194)
TMT-A	0.005 (0.913)			
		−0.167 (0.000)		
			0.098 (0.031)	
				0.106 (0.019)
		−0.159 (0.001)	0.053 (0.354)	0.054 (0.347)
TMT-B	−0.121 (0.006)			
		−0.098 (0.032)		
			0.112 (0.010)	
				0.140 (0.001)
	0.074 (0.099)	−0.083 (0.065)	0.116 (0.037)	0.056 (0.308)

*Standardized coefficients β (p-value) are expressed. Adjusted for age, sex, school years, and apoE 4 status. In the tests that revealed a significant association with DM, further adjustments were made for any significantly associated factors among HOMA-IR, gait speed, and HbA1c. HOMA-IR values were associated with logical memory II. Gait speed was associated with logical memory I, semantic fluency, letter fluency, digit symbol, TMT-A, and TMT-B. HbA1c values were associated with semantic fluency, digit symbol, TMT-A, and TMT-B. DM was associated with MMSE, semantic fluency, digit span, digit symbol, TMT-A, and TMT-B. In the tests that revealed a significant association with DM, further adjustments were performed for any significantly associated factors among HOMA-IR, gait speed, and HbA1c. For semantic fluency, TMT-A, and TMT-B, a further adjustment for gait speed and HbA1c diminished the statistical significance of DM. For the digit symbol test, further adjustment for gait speed and HbA1c attenuated the statistical significance of DM*.

In the tests that revealed a significantly association with DM, we performed further adjustment for any factors that were significantly associated with cognition among HOMA-IR, gait speed, and HbA1c. For semantic fluency, TMT-A, and TMT-B, further adjustments for gait speed and HbA1c diminished the statistical significance of DM. For the digit symbol test, further adjustments for gait speed and HbA1c attenuated the statistical significance of DM (Table 4).

## Discussion

Our participants with DM had comparable muscle mass but significantly slower gait speed compared to those without DM. Slow gait speed and hyperglycemia have been associated with cognitive dysfunction, mainly in the executive function domain, whereas IR is associated with memory impairment. The participants with DM had lower general cognition and executive function. The executive dysfunction in the DM group seemed to be partly explained by hyperglycemia and/or slow gait speed.

DM has been reported to be a risk factor for lower muscle performance (Volpato et al., 2012; Kalyani et al., 2013). Several mechanisms are involved in DM-related sarcopenia (Umegaki, 2016). Although, muscle mass is not necessarily decreased in DM, muscle performance is impaired in DM (Guerrero et al., 2016). Our present finding that DM subjects had slower gait speed agreed in part with these observations.

In the current study DM subjects had significantly lower scores in general cognition, and in the executive function domain; they also had significantly lower scores in MMSE, semantic fluency, digit span, digit symbol, TMT-A, and TMT-B. It has been established that older individuals with DM have impaired cognition. In addition, a previous meta-analysis showed that executive dysfunction is common among individuals with DM (Palta et al., 2014), and general cognition measured by the MMSE was found to reduced in a DM group compared to the non-DM controls (Mogi et al., 2004).

We also observed that lower gait speed was associated with executive dysfunction. In fact, lower gait speed was associated with lower performance in a wide range of tests that measure executive function. Systematic reviews have confirmed the association between lower gait speed and executive dysfunction (Kearney et al., 2013; Demnitz et al., 2016), although the mechanism underlying this association remains to be elucidated. Because the design of our present investigation was cross-sectional, the cause-result relationship is unclear. However, several possible explanations for this relationship can be suggested. Walking requires complex coordination of the extremities, and thus executive dysfunction may slow the gait speed through impairments of attention, judgment, and/or coordination. Alternatively, impaired gait function may limit social interactions and engagement in leisure activities—both of which changes have been associated with cognitive decline—and it may increase the risk of depression (Shankar et al., 2013). As another possible explanation of the association between lower gait speed and executive dysfunction, recent research has revealed that muscle secretes several factors called myokines. Myokines regulate the lipid and glucose metabolism, and they are associated with insulin resistance through the control of inflammation (Eckardt et al., 2014).

Another hypothesis is that shared causes of brain deficits may induce both executive dysfunction and slow gait speed. Subcortical ischemic vascular diseases are very common in older individuals and have been associated with both executive dysfunction (Jokinen et al., 2009), and slow gait speed (Rosano et al., 2005; Kreisel et al., 2013). A recent study also reported that amyloid beta deposition was associated with slow gait (Nadkarni et al., 2017). Alternatively, inflammation may be a shared pathology in both cognitive and physical dysfunction.

Systemic insulin resistance has been suggested to be associated with brain IR (Kullmann et al., 2012). Therefore, the association between systemic IR and cognitive dysfunction may be due to an impairment of insulin signaling in the brain. Insulin signaling in the brain inhibits glycogen synthase kinase 3 (GSK-3), which phosphorylates tau to induce neurofibrillary tangles (Kandimalla et al., 2016). A deficiency of insulin effects in the brain is thought to accelerate AD pathology. The first cognitive symptom in the disease process of AD is memory loss. The association that we observed between memory decline and IR in the present study's population partially agreed with the hypothesis that IR accelerates AD-related pathology in the brain.

Our study also indicated that HbA1c is associated with the results of several cognitive tests, mainly for executive function. Blood glucose itself may contribute to the cognitive decline in individuals with DM. HbA1c is a marker of the average blood glucose level over a relatively short term, i.e., 1–3 months (Parrinello and Selvin, 2014). High blood glucose may affect cognition relatively quickly. Or, many individuals with DM may tend to have a stable blood glucose status, and HbA1c at a certain point may represent the blood glucose status over longer periods.

As shown in Table 4, DM was associated with impairment in several neuropsychological assessments in the present analyses. However, the statistical significance of the associations between DM and lower scores in cognitive tests were diminished (semantic fluency, TMT-A, TMT-B) or attenuated (Digit symbol) by the adjustments for gait speed and/or HbA1c when these parameters were also associated with cognitions. The cognitive impairment (mainly in executive function) was associated with physical performance or blood glucose levels, and these factors at least partly explained the association between DM and cognitive dysfunction, which may suggest that the underlying mechanisms of DM-related cognitive dysfunction involve these factors—i.e., low physical function measured by slow gait speed and hyperglycemia represented by higher HbA1c.

An interventional study indicated that physical intervention was more effective in individuals with DM compared to non-DM subjects (Espeland et al., 2016). The current study was a baseline analysis of an interventional study which was originally designed to assess the contribution of physical exercise to cognitive preservation with physical exercise. The results regarding the effects of interventions in our cohort will be reported in the near future.

A major limitation of the present study was its cross-sectional design; because of this design, the cause-effect relationships were unclear. In addition, we analyzed the baseline data obtained in a randomized control trial with exercise intervention. Ideally, it would have been helpful to determine whether physical or metabolic improvement by exercise can lead to cognitive improvement. Other limitations of the study include the fact that the diagnosis of DM was based mainly on the participants' self-report. Finally, we did not fully account for medications that could potentially affect IR, since this information was lacking in the baseline database used.

All three factors that were significantly associated with cognitions in the current study—IR, gait speed, and hyperglycemia—are modifiable. Indeed, exercise may improve all three factors. In addition, antidiabetic agents improve hyperglycemia, and some medicines may improve IR. The above three factors were differently associated with different cognitive domains. A comprehensive approach to all three factors is warranted.

In conclusion, we assessed the associations among IR, hyperglycemia, DM, sarcopenia, and cognition. Our results showed that DM subjects had lower cognitive function compared to non-DM subjects. Among the factors assessed in the current study, IR was associated with memory impairment, while slow gait speed and hyperglycemia were associated with a wide-range of cognitive domains, but primarily executive function. The significance of the association between DM and cognitive dysfunction was attenuated upon adjustment for slow gait speed and hyperglycemia, which may suggest the involvement of these factors in the underlying mechanism of DM-related cognitive dysfunction. Further clarification of the mechanisms underlying these associations will contribute to the prevention of cognitive decline in the elderly.

## Author contributions

HU: design of the study, statistical analysis, manuscript writing, TM: design of trial, protocol development, data collection. KU: design of the study, data collection. XC, HS: review of study design and study supervision. TH: choice of measures and creation of intervention program. MK: design of trial, analysis plan, and critical revision of the manuscript. All authors have read and approved the final manuscript.

### Conflict of interest statement

The TOPICS study is an investigator-initiated study funded by the Toyota Motor Corporation. The authors declare that the research was conducted in the absence of any commercial or financial relationships that could be construed as a potential conflict of interest.
